# Fine-grained data reveal segregated mobility networks and opportunities for local containment of COVID-19

**DOI:** 10.1038/s41598-021-95894-8

**Published:** 2021-08-19

**Authors:** Chao Fan, Ronald Lee, Yang Yang, Ali Mostafavi

**Affiliations:** 1 Zachry Department of Civil and Environmental Engineering, Texas A&M University, College Station, TX 77843 USA; 2 Department of Computer Science and Engineering, Texas A&M University, College Station, TX 77843 USA

**Keywords:** Civil engineering, Sustainability

## Abstract

Deriving effective mobility control measures is critical for the control of COVID-19 spreading. In response to the COVID-19 pandemic, many countries and regions implemented travel restrictions and quarantines to reduce human mobility and thus reduce virus transmission. But since human mobility decreased heterogeneously, we lack empirical evidence of the extent to which the reductions in mobility alter the way people from different regions of cities are connected, and what containment policies could complement mobility reductions to conquer the pandemic. Here, we examined individual movements in 21 of the most affected counties in the United States, showing that mobility reduction leads to a segregated place network and alters its relationship with pandemic spread. Our findings suggest localized area-specific policies, such as geo-fencing, as viable alternatives to city-wide lockdown for conquering the pandemic after mobility was reduced.

The COVID-19 pandemic has caused enormous public health and economic impacts^1^. Close proximity contact is one of the major drivers of the pandemic^2^. In the absence of effective vaccines and drugs, many countries have enacted non-pharmacologic measures, such as travel restrictions, non-essential business lockdown, and stay-at-home orders to limit human mobility, intending to reduce cross-region epidemic transmission^3,4^. While mobility reduction measures are deemed an effective approach to disease containment^5^, empirical knowledge of the relationship between heterogeneous mobility reductions and pandemic spread in different stages is scarce^6^.

Multiple studies^7–9^ that have theoretically modeled and simulated the course of the COVID-19 pandemic with restricted mobility scenarios have demonstrated that mobility reduction could effectively delay the spread of SARS-CoV-2 across locations^10^. But as actual population movements reduced heterogeneously^11^, the results from theoretical models and simulations would be less useful if their assumptions on mobility reduction were inconsistent with the empirical evidence. This limitation may lead to an overestimation of the effectiveness of existing containment measures based on mobility reduction and create barriers to generate complementary policies^12^. The main goal of mobility reduction is to achieve sufficient spatial isolation among different regions of cities and their residents^13^.

In this study, we use fine-grained mobile phone data, which contain more than 1.15 billion dwelling points for 3.7 million anonymized devices from 21 of the most infected counties in the U.S., such as Harris County, San Francisco County, counties in New York City; and the city of Washington, D.C. based on the cumulative number of infected cases in March 2020. To characterize the spatial reach of people, analogously to ref.^14^, we computed the radius of gyration ($r_{g}$) for each individual device and the lengths of trips. Comparing to the baseline in January 2020, consistent with previous research^15^, we observed disproportionate reductions in mobility across distance categories during the outbreak of the COVID-19 pandemic. The variation in mobility reductions alters the way spatial census block groups (CBGs) are connected in a city and could have an influence on the trajectory of the pandemic. Our finding—that CBGs are connected in a sparser mobility network—suggests that a substantial spatial divide is achieved due to mobility reductions, and such a divide provides opportunities for area-specific containment policies, such as geo-fencing or ZIP-code lockdown, to further isolate CBGs with a greater incidence of infections.

## Results

**Figure 1:**
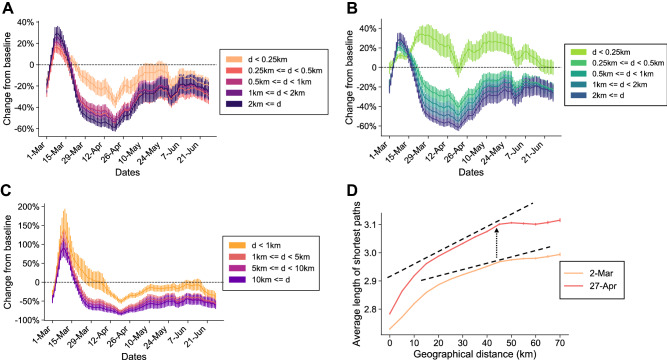
Empirical assessment of urban mobility changes during the COVID-19 pandemic across 21 metropolitan counties in the United States. (**A**) Relative changes in the number of trips (7-day average) based on distance categories. (**B**) Relative changes in the size of population (7-day average) based on the categories of the radius of gyration. (**C**) Relative change in number of edges based on the categories of the distance between two people's CBGs. (**D**) Average length of shortest paths in urban mobility networks on two typical dates as a function of geographical distance between two CBGs.

Figure 1A shows the relative changes in the number of trips ($n_{t}$) from the baseline for 21 metropolitan counties in the United States from March 1 to June 27, 2020. Note an abrupt decline, especially for the long-distance trips, occurring at the end of March and during April, when the stay-at-home orders were implemented. The largest reduction (around -60% below the baseline for long-distance trips and -40% below the baseline for short-distance trips) occurred in late April. The discrepancy between short- and long-distance trip patterns, slightly declining in the re-opening period, remained stable over the course of the pandemic.

Similarly, population with large $r_{g}$ substantially decreased, leading to an increase in population with the smallest $r_{g}$ (Fig. 1B). This result provides evidence for the main outcome of stay-at-home orders, which was reducing the spatial reach of residents with the result of a slowdown in the spread of the virus. Such a pattern of mobility change also influences social contact activities across CBGs of cities. Figure 1C presents the relative change in the number of edges for the distances between the CBGs where two connected individuals originate. Notably, there is a sharp decrease (close to − 100%) in the number of edges connecting people from two CBGs distant from each other in March and April, while the neighbor contact activities (the edges connecting people from the same or close CBGs) did not change as much (about − 50%) and returned to the baseline values rapidly during re-opening.

**Figure 2:**
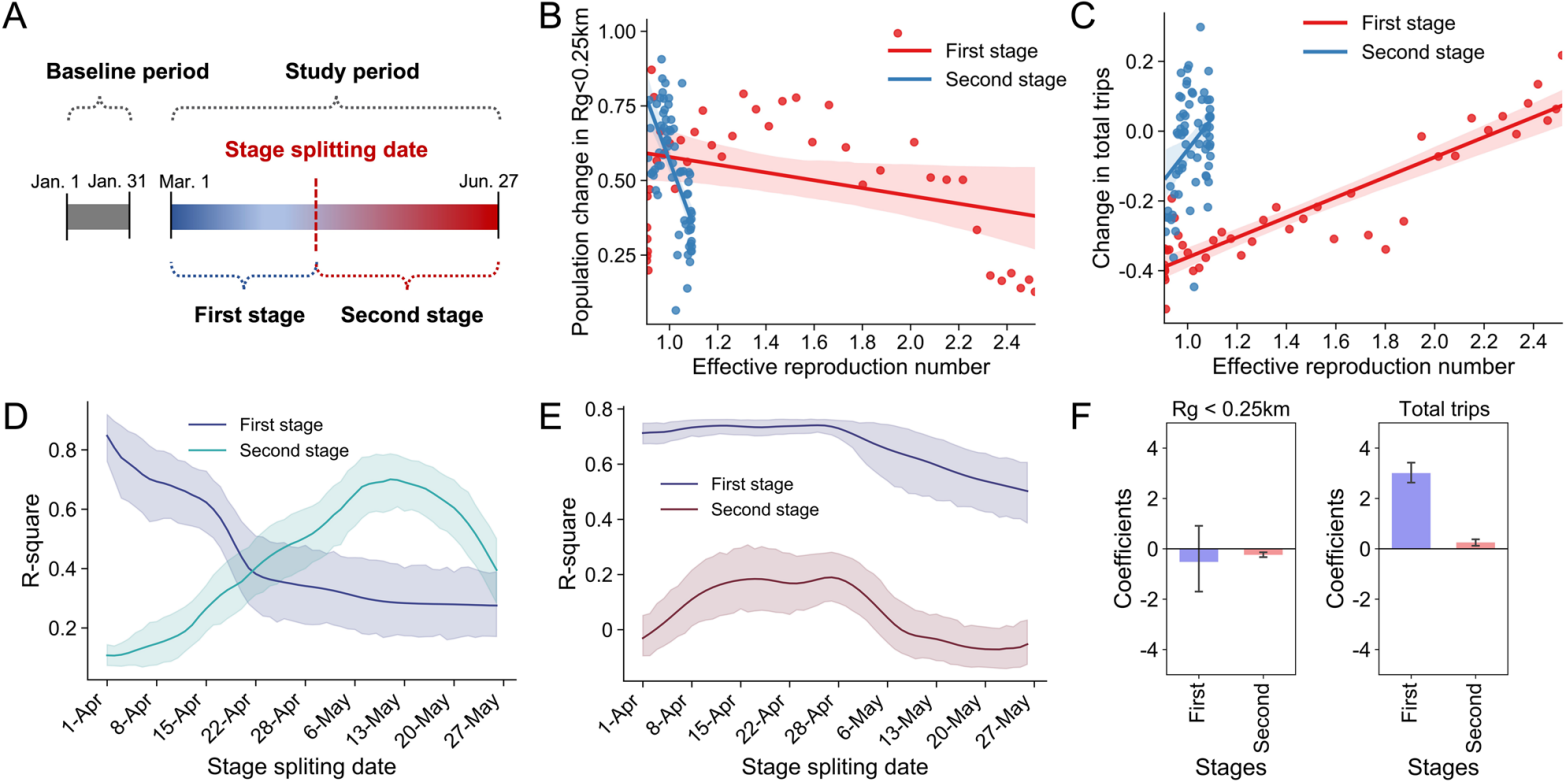
Results of stage﻿ splitting and dynamic relationships between mobility change and pandemic spread. (**A**) An illustration of stage splitting. (**B**) Example (Oakland County) of relationship change between effective reproduction number and population in smallest $r_{g}$. The stage splitting date is April 20, 2020. (**C**) Example of relationship change between $R_{t}$ and number of trips. (**D**) The $R^{2}$ for the OLS regression between the number of people in the category of the smallest radius of gyration and $R_{t}$ in two stages based on stage-splitting dates in April and May. (**E**) The $R^{2}$ for the regression between the number of total trips and $R_{t}$ in two stages based on stage-splitting dates in April and May. (**F**) The coefficients $\beta_{1,i}$ values in two different stages for two metrics. The splitting date in this example is April 20, 2020.

The split between changes in short- and long-distance movements provides insights regarding the change in mobility networks. Figure 1D presents the consequence of the mobility change pattern reflected in the transformation of mobility networks. We observed a striking increase in the length of shortest paths across CBGs from March 2 (before implementing orders) to April 27, 2020 (when mobility metrics bottomed). As geographical distances between pairs of CBGs grew, the average length of shortest paths increased more rapidly on April 27. This result explicitly shows that the mobility network on April 27 was more segregated than that on March 2. This empirical evidence uncovers the main way mobility reductions help with pandemic mitigation: through division of spatial units (CBGs) and their residents. This isolation could further alter the relationship between urban mobility and pandemic spread (Fig. 2B,C).

To further shed light on the dynamic interplay between urban mobility reduction and pandemic spread, we first estimate the daily effective reproduction number ($R_{t}$) as a proxy indicator for pandemic spread in each county. Then we select a date in April or May to split the study period into two stages (Fig. 2A). These two stages represent the time at which the relationship between mobility and pandemic spread changed. Here, we consider that all the dates in April and May 2020 could be the splitting dates. Hence, we selected each date as the stage splitting date and evaluated the R-squares of the model in two stages based on the selected dates. We illustrate the results generated by ordinary least squares (OLS) regressions for the association between $R_{t}$ and relative change in population size in the smallest $r_{g}$ (Fig. 2D) and relative change in $n_{t}$ (Fig. 2E). We find that when the dates in the beginning of April are selected as stage splitting dates, the relationship between $R_{t}$ and relative change in population size in the smallest $r_{g}$ (Fig. 2D) was significantly strong in the first stage from March 1 to the beginning of April, while the relationship was particularly weak in the second stage from the beginning of April to the end of May. This observation allows us to determine dates when the pandemic situation shifts the relationship between movement radius and effective infections. In the application of our findings, the difference of the R-squares at two stages is an important metric to determine the stage splitting date.

In addition, we observe that including more data in the first stage weakens the associations between the two metrics. The population size in the smallest $r_{g}$, however, can still explain more than 60% of the variation of the $R_{t}$ in the second stage. The number of trips does not maintain its strong association with the pandemic spread (Fig. 2E). This result could be because the number of trips increased during the second stage, but the division among the spatial units, to some degree, remained. Furthermore, Fig. 2F shows that the coefficients of the regression models changed, in particular, for the number of trips in the second stage. Since $R_{t}$ was in general larger than 1, the segregation in mobility networks is not sufficient for conquering the disease. This result offers a need of localized area-specific measures, such as geo-fencing and ZIP code-level lockdown, to not only maintain short-distance movements and spatial divide, but also to deeply restrict activities of populations in areas with a more extensive number of reported cases.

## Discussion and concluding remarks

The results provide an empirical assessment to uncover how the public reduces movements in response to the COVID-19 pandemic and consequences on the change of mobility networks and the association with pandemic spread. We found that long-distance movements have reduced substantially, while short-distance trips changed slightly. People started moving in a small radius of gyration in response to the pandemic and subsequent stay-at-home orders. Such heterogeneous mobility reduction pattern greatly increased the length of shortest paths across CBGs and subsequently caused the mobility network of CBGs to be sparser and more segregated. This segregation in the mobility network of CBGs was the main outcome of mobility reduction policies. In this stage, CBGs were locally connected, instead of globally and densely connected as in normal conditions. Hence, the relationship between urban mobility and pandemic spread was reconfigured.

According to the findings in this study, we could summarize that the purpose of mobility reduction policies is to create spatial segregation by increasing the lengths of shortest paths in networks and reducing the spatial reach of larger populations at all scale, from global, to national, to city scale. This division in the spatial structure of urban mobility networks provides opportunities for localized area-specific policies for pandemic containment while the implementation of city-wide lockdown with reduced total number of trips would not be economically feasible. More localized containment such as geo-fencing, drawing a virtual perimeter that marks the limit of permitted scale of movement for residents, or ZIP code-level lockdown, could have a greater impact on pandemic containment when mobility networks are segregated. Specifically, clusters of CBGs with a large number of infections could be restricted in their CBGs. Other CBGs could lift their restrictions on local business and outdoor activities to recover the economy of cities.

As a first step to the long-standing question for the relationship between urban mobility and disease spread, this report provides the potential of the novel data to initiate new avenues of research in policy making for pandemic mitigation. Multiple directions could further expand our research. For example, considering the time lags between two variables in autocorrelation analysis would be helpful for predictive tasks such as predicting the number of cases in the future. In addition, evaluating the proposed mitigation strategies such as geofencing using epidemiological simulation could also be helpful to validate the effectiveness of the strategies.

## Methods

This study utilized anonymized data provided by Veraset, Inc. Using data from 21 of the most highly infected metropolitan counties from January 1 through June 27, 2020. The data set contains anonymized device IDs, the timestamps and precise geographical coordinates of dwelling points. The dwelling points, also called stop points, in the anonymized mobile phone data shared by the data compony are defined as the points where the devices spent at least 5 min. It is obtained from granular device location points by clustering the points which are spatially and temporally proximate. In addition, we labeled the dwelling points with points of interest (POIs) and CBGs. The locations of POIs were provided by SafeGraph, Inc. The geographical boundaries of CBGs were provided by The Centers for Disease Control and Prevention (CDC) (2018 documentation). Before performing the analyses, we first filtered out the data points of the devices that only appear for a couple of days in the date set. The data points of the devices that have records in at least three-quarters of a month are included in the analyses of this study. Hence, the population size in the analyses of this study is consistent during the study period.

A trip of a device/user is defined as the movement from one dwelling point to another^16^. The number of trips is computed using the movements of devices between two dwelling points. Here, we considered all the trips including the trips repeated by a device in a day. That means, if one device moved between two points twice in a day, we consider these as two trips. We classified the trips into five distance categories based on the geographical distance between the starting and ending dwelling points. The baseline data for the number of trips is the average daily number of trips at the same day of the week in January 2020 in each county in order to take the weekly pattern into account^17^. The change of the population size ($c$) is calculated by:

1 $$c = \frac{{p_{i} - \overline{p}_{s} }}{{\overline{p}_{s} }}$$

where $p_{i}$ represents the population size in a category of radius of gyration or the number of trips on date $i$ ranging from March 1 to June 27, 2020, and $\overline{p}_{s}$ represents the average population size in the corresponding category of radius of gyration or the number of trips in the same day of week in January 2020.

Consistent with the ref.^14^, we computed the radius of gyration entered in the trajectory’s centre of mass for each unique device and computed daily population size in five radius categories. The individual devices’ radius of gyration in this study is defined as the characteristic distance traveled by the device during a day^14^. In this study, we adopted the method from prior influential research^14^ to calculate the radius of gyration for each individual device:

2 $$r_{g} = \sqrt {\frac{1}{n}\mathop \sum \limits_{i = 1}^{n} \left( {\mathop{r}\limits^{\rightharpoonup} _{i} - \mathop{r}\limits^{\rightharpoonup} _{cm} } \right)^{2} }$$

where $\mathop{r}\limits^{\rightharpoonup} _{i}$ represents the $i = 1, 2, \ldots , n{ }$ locations recorded for the device, and $\mathop{r}\limits^{\rightharpoonup} _{cm}$ is the trajectory’s center of mass and can be calculated by $\mathop{r}\limits^{\rightharpoonup} _{cm} = 1/n\mathop \sum \limits_{i}^{n} \mathop{r}\limits^{\rightharpoonup} _{i}$. Since the locations of the devices are recorded using geographical coordinates which are composed of latitudes and longitudes, we can calculate the radius of gyration using^18^:

3 $$r_{g} = \sqrt {\frac{1}{n}\mathop \sum \limits_{i = 1}^{n} \left[ {2r \times \sin^{ - 1} \left( {\sqrt {\sin^{2} \left( {\frac{{\phi_{i} - \phi_{cm} }}{2}} \right) + \cos \left( {\phi_{cm} } \right)\cos \left( {\phi_{i} } \right)\sin^{2} \left( {\frac{{\lambda_{i} - \lambda_{cm} }}{2}} \right)} } \right)} \right]}$$

where $r$ is the radius of the earth, $\phi_{i}$ and $\phi_{cm}$ are the latitudes of the location $i$ and the center of mass respectively, and $\lambda_{i}$ and $\lambda_{cm}$ are the longitudes of the location $i$ and the center of mass respectively. There are five categories for the radii of gyrations of the devices: $r_{g} < 0.25{ }km$; $0.25{ }km \le r_{g} < 0.50{ }km$; $0.5{ }km \le r_{g} < 1{ }km$; $1{ }km \le r_{g} < 2{ }km$; and $2{ }km \le r_{g}$. The category of the smallest radius of gyration means the category of $r_{g} < 0.25{ }km$.

To construct mobility networks, we first represented the trips between CBGs as a weighted network ${\mathcal{G}}\left( {V,E,W} \right)$, where $V$ represents a set of nodes (CBGs), $E$ represents a set of edges, and the weight ($w_{ij} \in W$) of an edge ($e_{ij}$) between CBGs $i$ and $j$ is the number of trips. The average shortest path metric is used to measure the connectedness of the nodes (census block groups) in the networks by taking into account the flow of population across different nodes. We consider two nodes to be “close” to each other when they are connected by a large flow value1. Since the analysis focuses on examining epidemic spread, the greater the population flow between two areas, the closer the two areas in terms of the ability of the virus to spread. Hence, we defined the distance between two CBGs in the mobility network as the inverse weight ($1/w_{ij}$) along the edge connecting these two CBGs. Then we adopted Dijkstra's algorithm^19^ to calculate the shortest path length between each pair of CBGs.

We employed an ordinary least squares regression model^20^ to estimate the relations between county $i$'s $R_{t}$ values (${\varvec{y}}_{i}$) and mobility metrics (${\varvec{x}}_{i}$). Since the effective reproduction numbers and mobility metric values are time series, they are represented by vectors in the model. The OLS regression takes the following form:

4 $${\varvec{y}}_{i} = \beta_{0,i} + \beta_{1,i} {\varvec{x}}_{i} +_{i}$$

where $\beta_{0,i}$ and $\beta_{1,i}$ are parameters and $\epsilon_{i}$ is an error term. We conducted the regression for each mobility metric and stage separately. Equation (4) above is a general form of the regression models. The ordinary least squares regression model employed in this study is used to illustrate the relationship between two variables. We do not aim to use this model for prediction. R-square is one of the most commonly adopted metric to evaluate the performance of the model, referring to existing literatures^21^. To make the process straightforward, therefore, we used R-square to indicate the relationships between the dependent and independent variables in this study.

## Data Availability

The fine-grained anonymized movement data are available from Veraset, Inc. (provided upon request submitted at https://www.veraset.com). The CBG data is provided by CDC (https://www.atsdr.cdc.gov/placeandhealth/). The geographical locations of POIs are provided by SafeGraph (freely provided upon request submitted at https://www.safegraph.com/covid-19-dataconsortium). The COVID-19 case data were obtained from https://github.com/nytimes/covid-19-data. The $R_{t}$ for each county was calculated using the method on rt.live. The code used to run the analyses and models is available on Github https://github.com/urban-resilience-lab.
